# Time to loss of physical integrity of attractive targeted sugar bait (ATSB) stations in Western Province, Zambia: a survival analysis

**DOI:** 10.1186/s12936-025-05316-7

**Published:** 2025-03-15

**Authors:** Refilwe Y. Karabo, Masuzyo H. Mundia, Mwansa Mwenya, Kochelani Saili, John Miller, Kafula Silumbe, Irene Kyomuhangi, Joseph Wagman, Javan Chanda, Erica Orange, Busiku Hamainza, Angela F. Harris, Julian Entwistle, Laurence Slutsker, Thomas R. Burkot, Megan Littrell, Thomas P. Eisele, Ruth A. Ashton, Joshua Yukich

**Affiliations:** 1 Department of Tropical Medicine, Center for Applied Malaria Research and Evaluation, Tulane University Celia Scott Weatherhead School of Public Health and Tropical Medicine, New Orleans, USA; 2 PATH, Lusaka, Zambia; 3 Lancaster University, Lancaster, UK; 4 PATH, Seattle, USA; 5 National Malaria Elimination Centre, Lusaka, Zambia; 6 IVCC, Liverpool, UK; 7 Independent Consultant, Atlanta, GA USA; 8 Australian Institute of Tropical Health and Medicine, Cairns, Australia

**Keywords:** Malaria, Attractive targeted sugar bait, Survival

## Abstract

**Background:**

Attractive targeted sugar baits (ATSBs) are a potential addition to an integrated vector management strategy against malaria. ATSB stations, which include a sugar bait and an ingestion toxicant, could potentially be deployed to attract-and-kill mosquitoes and thereby prevent malaria transmission. The physical durability of these stations is likely to be an important factor in decisions around future use strategies. This study measured the duration of physical integrity of the ATSB Sarabi v1.2 stations used in Western Zambia, as part of a Phase III cluster RCT.

**Methods:**

ATSB stations were installed and followed as a cohort on the external walls of selected sleeping structures in households in trial clusters (10–11 per cluster). Monthly visits were made to assess the presence and condition of the ATSBs from November 2022 to June 2023. A rolling cohort approach was used, whereby new ATSB stations were used to replace those which failed or were lost-to-follow-up, and these were subsequently enrolled in the cohort. Information on structure construction and ATSBs location on the walls was also collected. Median ATSB survival and associated factors were analysed with Kaplan–Meier curves and Cox-Proportional hazard models.

**Results:**

Including replacements, a total of 1107 ATSBs were installed across 304 sleeping structures in 206 households, and 5696 ATSB-visits were made. Common types of damage observed were holes/tears, mold, and leakage of bait. While the median survival time for the devices was 5 months (149 days) for all stations in the study, the median survival time was longer than the transmission season for stations installed in locations well protected by the roof (> 218 days). ATSB station survival was longer when installed on structures with thatched roofs compared to iron-sheet roofs (HR 0.37, 95% CI 0.26–0.47, p < 0.001), and where there was “excellent protection” (HR = 0.36, 95% CI 0.25–0.49, p < 0.001), compared to “no protection”.

**Conclusions:**

Study results suggest that the majority of ATSB stations deployed in this setting will remain intact for a 7-month seasonal deployment period if stations are installed in locations protected from weather elements, such as underneath the overhang of thatched roof. Further research is needed to understand factors that influence the physical integrity and efficacy of ATSB stations in addition to those observed in this study.

## Background

Almost eighty percent of the global reduction in *Plasmodium falciparum* malaria cases since the year 2000 has been attributed to vector control (largely long-lasting insecticidal nets and indoor residual spraying) [1]. The reduction in malaria cases has recently stalled, highlighting the need for new vector control tools to further reduce malaria transmission and, ultimately, achieve elimination and eradication of malaria.

Plant sugars are an essential energy source for both male and female mosquitoes. This requirement can, in theory, be targeted by providing an artificial sugar source as an attractant to lure and, if combined with an insecticide, to kill mosquitoes. This approach to vector control has been called Attractive Targeted Sugar Baits (ATSB) and follows an attract-and-kill paradigm of vector management. ATSB stations are a promising innovation and a potential addition to existing vector control tools [2, 3]. ATSB stations thus differ from the existing recommended vector control methods that target indoor human blood feeding (insecticide-treated nets) and resting behaviours (indoor residual spraying, IRS), by targeting a different behavioural component of the mosquito life-cycle. In 2017, ATSB stations were shown to reduce *Anopheles* mosquito densities in field settings in Mali [4, 5]. A phase III cluster randomized control trial (cRCT) was conducted in Western Zambia to investigate the efficacy of the Sarabi v.1.2 ATSB stations (manufactured by Westham Ltd., Hod-Hasharon, Israel) in reducing clinical malaria incidence.

The sustained effectiveness of many vector control products, when deployed, depends on their physical integrity and, ultimately, their durability (ability to resist wear and remain effective over time), though the specifics of what is meant by durability varies with product class and intervention type. For example, long-lasting insecticidal net (LLIN) durability has been previously defined to include persistent insecticidal effectiveness, physical durability, and attrition [6–8]. In the context of IRS, insecticide residual efficacy is used to characterize intervention durability or persistence in the field [9]. Due to the novelty of the Westham Sarabi v.1.2 ATSB stations, there is limited understanding of their durability, and their bio-efficacy and effectiveness under deployment conditions. In a recent study by Mwaanga et al*.* [2], the bio-efficacy of ATSB stations was measured for ATSB tools that remained physically intact during field deployment. The results indicated that ATSB stations that remain physically intact could also remain bio-efficacious following deployment for at least 7 months in the field. There are, however, currently no studies on the physical durability and survival of the ATSB products under routine deployment conditions.

Kyomuhangi et al. [10], report on trends in damage to ATSB stations during the cRCT. That study assessed the temporal and spatial trends in damage to ATSB stations, offering insights into the prevalence of overall damage and different damage types impacting these ATSB stations. Results showed temporal and spatial variation in overall damage and different damage types throughout the deployment period and across the trial site. Additionally, the likelihood and rate of damage to ATSB stations in this context were influenced by characteristics of the dwelling structures, as well as environmental factors.

This study seeks to ascertain the duration of physical integrity and time to attrition (median lifetime) of the Westham Sarabi v1.2 ATSB stations used during the Zambia cRCT, which are likely to be important components of the overall durability of ATSB. Factors that include the duration of physical integrity, bio-efficacy, and attractancy, as well as attrition, are relevant in this context. The aim of this study is to assess the median time that the ATSB stations remain deployed in the field in a selected sample before replacement due to deterioration in physical integrity, and do not go missing, which we describe as the survival time.

## Methods

### Study site

The cRCT trial was conducted in Kaoma, Luampa, and Nkeyema districts, in Western Province, Zambia, from November 2021 to June 2023. The study site is described by Arnzen et al*.* [11], details of the trial design are reported by Eisele et al. [12], and the ATSB intervention is described in detail by Orange et al*.* [13]. The three trial districts have a combined population of 246,785 (2022 population census) [14]. ATSBs were deployed seasonally during the main malaria transmission season in each year (two cycles) of the trial. This study was conducted during the second cycle (November 2022–June 2023). The climate of the study area is tropical, with a rainy season from approximately December through March [11], which supports arable and pastoral agricultural activities. The rainy season is followed by a peak in malaria transmission from April to May.

### ATSB stations

The ATSB Sarabi v.1.2 bait station (Westham Ltd., Hod-Hasharon, Israel) measures slightly over 24 cm X 30.5 cm. Each bait station has 16 cells filled with 72 g of date syrup-based bait, which acts as both the attractant and sugar source; dinotefuran (0.11% w/w) the active ingredient; and Bitrex^®^ (Johnson Matthey), a bittering agent to deter ingestion by humans. Dinotefuran, an ingestion toxicant, is a furanicotinyl (neo-nicotinoid) insecticide, that occupies and activates the nicotinic acetylcholine receptors of the mosquitoes [15]. The toxicant binds permanently to the insects’ nicotinic receptors and mimics the effects of acetylcholine, resulting in constant nerve stimulation, tremors, and eventually death [16]. ATSB station contents are contained between a white plastic backing and a black perforated membrane that allows the mosquito proboscis to penetrate and ingest the bait, while reducing the ability of non-target organisms to access the bait. It also allows for evaporation of volatile components in the bait, including water. The white plastic backing, and membrane are fused together in the 1 cm spaces between the cells. Further details of the ATSB station used in this trial are reported by Orange et al*.* [13].

### ATSB station installation campaigns

**Fig. 1:**
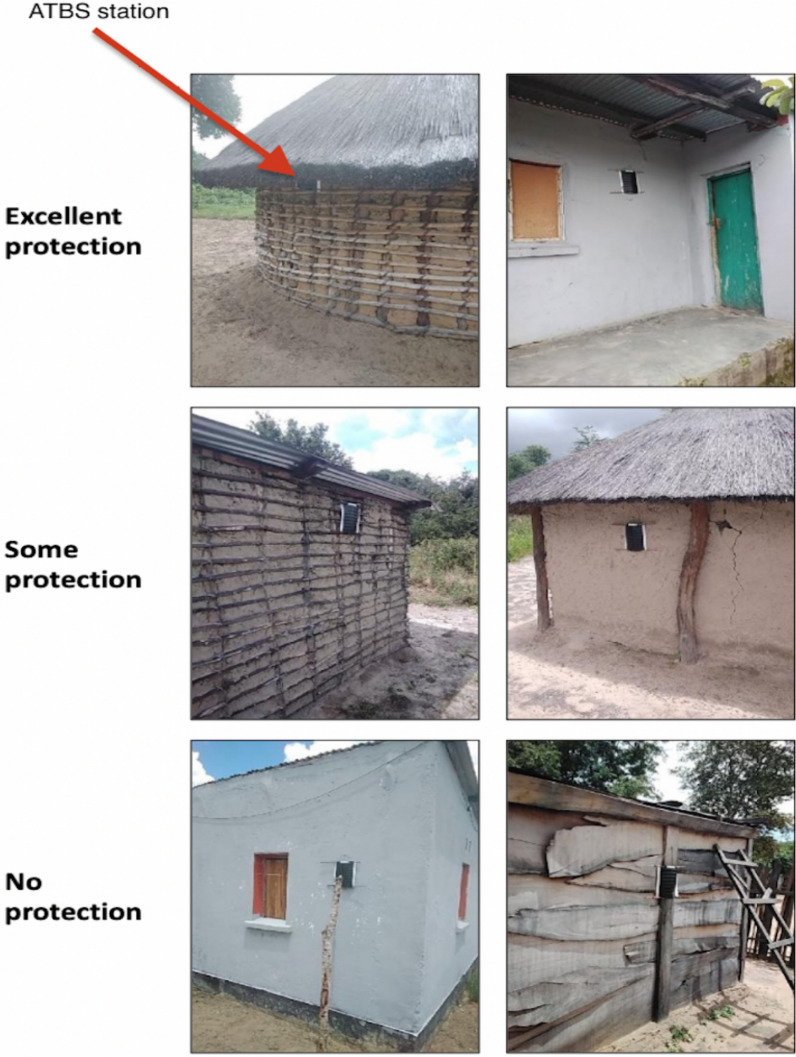
Examples of ‘protection levels’ of installation sites on structures within the study

ATSB stations for this durability study were installed on study households as part of the second year of the cRCT in November 2022 where all eligible structures in intervention areas of the trial had ATSB stations installed. Eligible structures that received ATSB stations were defined as those with a complete roof, at least three complete exterior walls, wall height measuring one meter or more, and structures not under construction (primarily sleeping structures and multi-use residential structures). Structures that were identified as shops, schools, churches, tobacco sheds, animal kraals, toilets, bathing shelters, and food storage shelters were not eligible for ATSB station installation. Two ATSB stations were installed on each eligible structure by trained community members (ATSB Monitors). ATSB stations were typically hung on opposite exterior walls of a structure unless adjacent walls offered better protection from rain, sun, and wind. ATSB stations were hung on walls using bamboo sticks inserted through holes on the top and bottom frames of the ATSB station. Strings or wires were wound around the bamboo sticks to attach the ATSB stations to anchored nails on the walls of the structures (see Fig. 1). Further details of ATSB station installation are available in Orange et al. [13].

### Study design and data collection procedures

A prospective, open cohort of ATSB was nested within the routine ATSB monitoring procedures of the ATSB trial. Selected ATSB stations installed during the trial installation campaign were enrolled in this study on a first visit by the research team shortly after placement on structures in late November to early December 2022. As a rolling cohort, additional ATSB stations installed and enrolled in the cohort either on new structures, or as replacements for damaged stations were also enrolled into this study.

Based on logistical considerations, 20 of the 35 intervention arm clusters were purposively selected for inclusion in this study. Ten or eleven households were purposively selected from each of the 20 clusters. This means the stations and structures were followed over time, but they were not sampled representatively. The enrolment visit occurred approximately 2 weeks after ATSB installation. On the enrolment visit (late November or early December 2022), the research team created sketch maps of the selected households, documented all the structures in each household, and recorded details of ATSB stations already present on structures from the ATSB installation campaign. Each ATSB station had a unique QR code which had been printed during manufacturing. Monthly visits were made to all enrolled households and structures. At each visit, the presence or absence of each ATSB station was confirmed, and the location was verified. ATSB stations were assessed according to the pre-defined damage criteria for replacement, including holes/tears, leaks, mold, dirt, and depletion [13]. The presence of mold that led to replacement was defined as fuzzy mold growth more than the size of the rubber/eraser end of a pencil or layer of mold that covered at least half of the ATSB station surface. Tears/holes were defined as one or more cells being fully open. Dirt that warranted replacement was described as having at least eight cells covered in dirt. ATSB trial staff also palpated the ATSB stations to check if each of the 16 cells on each station were depleted of the bait contents (including the attractant and ingestion toxicant) or not. ATSB stations that warranted replacement had eight or more cells flat or empty when checked with a gloved hand. A photograph of the ATSB station was also taken as evidence of its presence and condition. ATSB stations meeting the replacement criteria were replaced with a new station, and the QR code of the newly installed ATSB station was recorded and enrolled into the study.

During each study visit, the location, and characteristics of each ATSB station installation site were documented. These included the distance between the lower end of the ATSB station and the ground, the distance between the upper portion of the ATSB station and the roof (at the point where the roof and the exterior wall meet), as well as the length of the roof overhang (distance perpendicular to the outside wall). Information on the material used for construction of the structure’s wall and roof was also documented.

If an enrolled structure was demolished, burned, or collapsed, this was documented, and no further visits were made to it. If a new ATSB station-eligible structure was identified at an enrolled household during a study visit, the structure was enrolled. The existing ATSB stations would then be registered, or ATSB stations installed and registered if not already present. This study was embedded in the ATSB main cRCT monitoring programme; hence, the ATSB stations were also routinely assessed by ATSB monitors. This monitoring occurred once every 2 months or sooner if damage was reported by the household. It was therefore possible for ATSB stations to be visited more than once a month by the ATSB monitors as part of routine monitoring for the trial, and by the research team for the physical durability study. Routine monitoring visits used the same replacement criteria as this study to remove and replace damaged or lost bait stations.

Observations of ATSB stations were recorded using a standardized, XML form-based data collection system deployed on Android Mobile Phones (Commcare, Diamgi Inc., Cambridge, MA, USA).

### Sample size

The sample size was calculated following the Brookmeyer and Crowley method [17]. The median survival of an ATSB station was assumed to be 3 months, and the sample size was planned to allow for this to be estimated with a 95% Confidence Interval of 2.3–4.1 months. It was anticipated that this level of precision could be achieved if a minimum of two ATSB stations were installed and followed at each selected household; and a minimum of 10 households in 10 clusters were included. This resulted in a minimum of 100 households, and 200 ATSB stations, and potentially monitoring of more than 300 ATSB stations (including replacements) being followed for 6 months. Field logistics and funding permitted expansion of the study from 10 to 20 clusters, but retained a target enrolment of at least 10 households per cluster in theory, allowing for a more precise estimation of the study outcome.

### Data analysis

Data analysis was conducted using R programming language version 4.2.2 [18] and packages ‘survival’ [19], ‘survminer’ [20], ‘frailtypack’ [21], and ‘coxme’ [22].

### Outcomes and other measures

The main endpoint of the study was ATSB station meeting the criteria for replacement or having been removed. The outcome event was defined as occurring when the ATSB station met the replacement criteria on the monthly durability study visit or was not found where it was expected (either replaced by routine monitoring process or absent). The ATSB stations that had not experienced an outcome event continued to be followed and monitored in the subsequent visits to the end of the study or when lost-to-follow-up for other reasons, such as destruction or removal of the structure on which the station had been placed. Stations that did not experience an event were considered as right censored at their last visit. Right censorship occurs when the study ends with the ATSB still intact or when a subject, in this case, an ATSB station, is lost-to-follow-up due to the removal or destruction of the structure on which they were last deployed; thus, the ATSB could not be followed until its failure, and the true survival time could not be ascertained [23].

Time to event was measured in days until failure/event occurred or censoring. This was defined as the number of days between the ATSB station being enrolled in the study and the date a failure/event was recorded (ATSB station was found damaged, removed from its location or other reasons that met the pre-determined replacement criteria) or the ATSB stations follow-up time was censored.

Details of the ATSB station installation-location were used to generate an indicator of protection level with three classes. “Excellent protection” was defined as ATSB stations that were either reported as “tucked under the roof overhang” on observation or where the ratio of the measured roof overhang to the distance from the roof to the ATSB station was greater than one. “Some/moderate protection” was defined as ATSB stations that were reported as either being placed at level with the edge of the roof, or where the ratio of the measured roof overhang to the measured distance from the roof to the ATSB station was 0.5 or larger but less than one. “No protection” was defined as ATSB stations where the ratio of the roof overhang size to the distance from the roof to the ATSB station was < 0.5. Figure 1 shows photos of stations installed on study buildings as visual examples of these protection levels. The study also considered the height of the ATSB station above the ground. The measurements were categorized into four groups (< 100 cm, ≥ 100–150 cm, > 150–200 cm and > 200 cm between the ATSB station and the ground).

Kaplan–Meier survival estimates were used to calculate the overall median survival time and median survival time stratified by protection level. Cox-Proportional Hazard models were used to examine differences in survival by different roof materials, wall placement position, protection level and included the effect of study cluster via inclusion of shared frailties. Shared frailty methods account for unobserved random variation of the hazard due to shared risk of failure at the cluster level, which differs from that of the overall cohort. To determine if ATSB survival varied over time, Cox-Proportional Hazard models were extended to include a categorical variable of calendar month as a time-dependent covariate. Time-dependent models required data arranged by each ATSB visit, where calendar month represented the start of each interval between visits.

## Results

The study involved 304 eligible structures in 206 households for monthly visits. A total of 1107 ATSB stations across 20 clusters, with a range of 32–139 ATSB stations per cluster, were analysed. For monitoring purposes, 5,696 ATSB station-visits were made from November 2022 to June 2023 for this study (not including visits made by ATSB monitors). The maximum duration from the time of ATSB enrolment in the study to a final ATSB station-visit was 218 days.

Sixteen structures were excluded after enrolment as they were subsequently considered not eligible because they were no longer being used as sleeping structures, or because the structure had collapsed or burned after data collection had begun. During the study, 17 newly eligible structures were enrolled at participating households, with 34 new ATSB stations installed on these structures.

Among the enrolled structures, 35.9% (109/304) had wood walls with mud plaster, and half of all structures were roofed with iron sheets (152/304, 50%, Table 1). Table 2 indicates the distribution of all ATSB stations included in the study at any time, according to the wall type, protection level, and roof type of the structures they were installed on.

**Table 1:** Summary of structure characteristics

	N = 304 (%)
Wall type	
Wood with mud plaster	109 (35.9)
Cement block	72 (23.6)
Mud brick, covered^a^	31 (10.2)
Mud brick, uncovered	68 (22.4)
Wood plank	14 (4.6)
Thatch	10 (3.3)
Roof type	
Iron Sheet	152 (50)
Thatch	145 (47.7)
Cement	5 (1.6)
Wood plank	2 (0.7)

^a^Mud bricks are covered with mud or cement plaster

**Table 2:** Structure characteristics of installed ATSB stations

	Protection level of installed ATSB		
	Excellent protection (N = 621)	Moderate protection (N = 292)	No protection (N = 194)
Hung on wall type			
Wood with mud plaster	319	70	8
Cement block	20	115	126
Mud brick, covered^a^	65	39	6
Mud brick, uncovered	174	32	43
Wood plank	10	32	11
Thatch	33	4	0
Hung under roof type			
Iron sheet	100	281	173
Thatch	521	6	0
Cement	0	0	21
Wood plank	0	5	0

^a^Mud bricks are covered with mud or cement plaster

Overall, 48.6% of enrolled ATSB stations met the criteria for failure and reason for replacement during the study period (up to 218 days). The median time (of ATSB stations) from enrolment to replacement or loss by cluster ranged from 35 days to beyond the maximum 218 days of the study period. Nine of the 20 clusters (45%) had ATSB stations with median lifetime greater than the 218-days, indicating that in those clusters, more than half of the ATSB stations enrolled at the start of the study remained hanging and intact at the end of the study period.

The most common reasons for ATSB station replacement/event were holes/tears (24.3%), followed by mold (21.6%) and leaking (19.3%) (Table 3). Stations with two or more categories of damage contributed 2.6% of the observed reasons for replacement. One-hundred and eight (20.1%) enrolled ATSB stations were replaced by trained community members during routine monitoring visits between the scheduled visits of this study. Relatively few ATSB stations (5.6%) were missing or otherwise lost to follow-up.

**Table 3:** Reasons for ATSB replacement

	ATSBs N = 538 (%)
Damaged: torn	131 (24.3)
Damaged: mold	116 (21.6)
Damaged: leak	104 (19.3)
Damaged: depleted	30 (5.6)
Damaged: dirty	5 (0.9)
Damaged: more than one category	14 (2.6)
Absent/missing	30 (5.6)
Removed by community monitor^a^	108 (20.1)

^a^Separate from the research team in this study, ATSB monitors visited bait stations to perform assessments of bait station conditions and to remove and replace damaged bait stations during routine monitoring for the trial

A total of 569 (51.4%) of the 1107 ATSB stations were right-censored. This included ATSB stations not meeting replacement criteria at the final visit and those right censored during the course of the study (including those hung on structures that later collapsed, burned, or ineligible). A total of 611 ATSB stations that were tucked under roof overhang were provided with “excellent protection”, while 28 had “moderate protection”, and only 3 had “no protection” (Table 4). Further, Table 4 illustrates that ATSB stations that had “excellent protection" were placed under roof overhang with a significant size (median 50 cm), compared to those with minimal protection of the roof overhang, at a median of 30 cm “moderate protection”, and 25 cm “no protection”, respectively.

**Table 4:** Summary of protection ratios and components

	Excellent protection (N = 621)	Moderate protection (N = 292)	No protection (N = 194)
Qualitative description			
Tucked under roof overhang	611	28	3
Level with edge of the roof	0	38	6
Lower down the wall from roof overhang	10	226	185
Roof overhang			
Median	50	30	25
IQR	30	30	10
Installation height			
Median	120	150	156
IQR	30	39	30

**Fig. 2:**
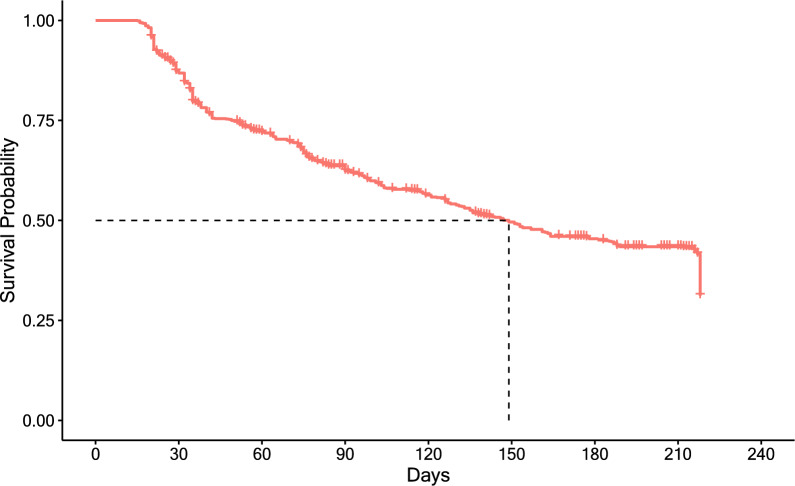
Kaplan–Meier curve of ATSB survival time in days for pooled data from all 20 clusters

**Fig. 3:**
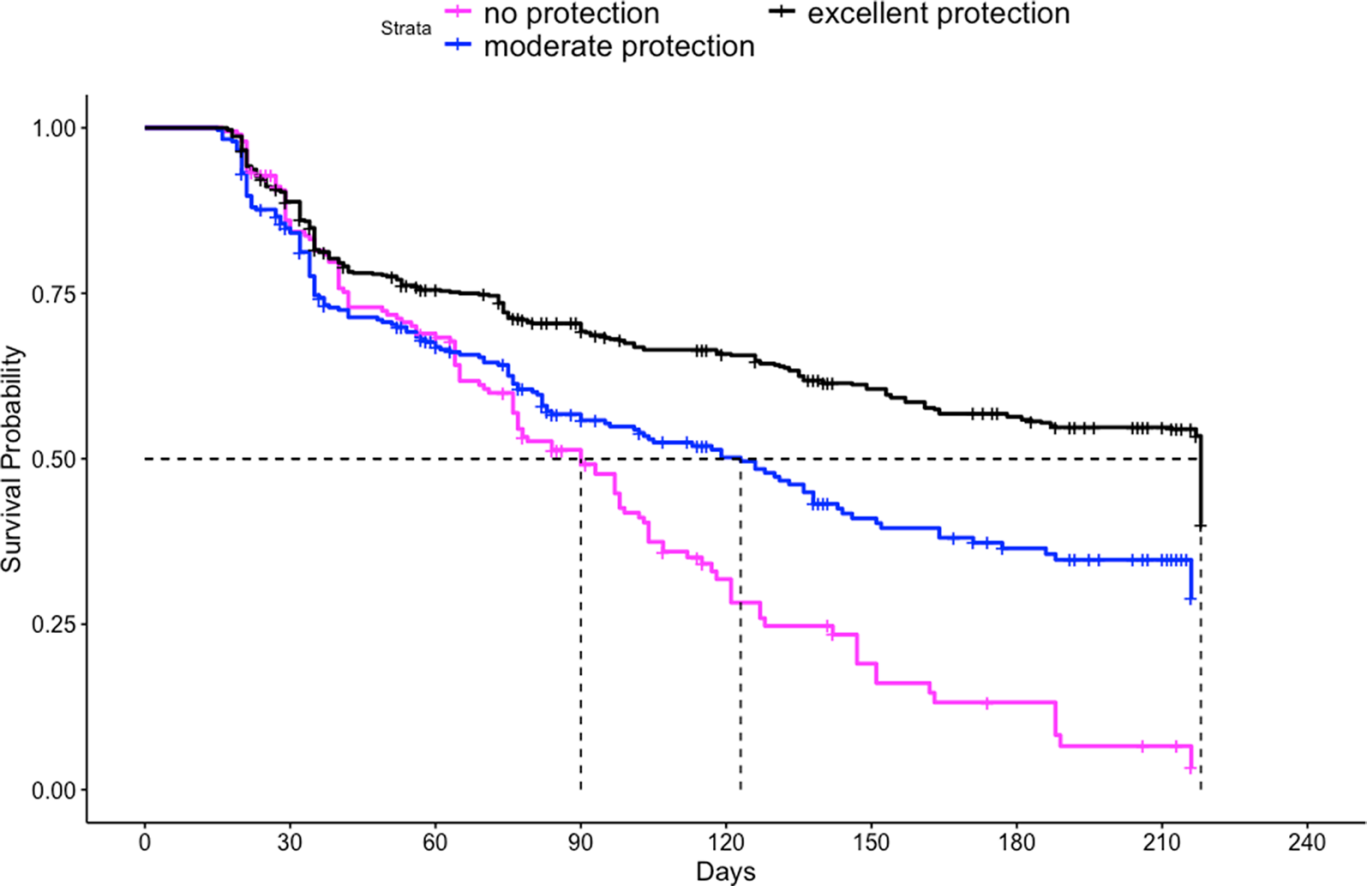
Kaplan–Meier curves of ATSB survival time in days by level of ATSB protection

**Fig. 4:**
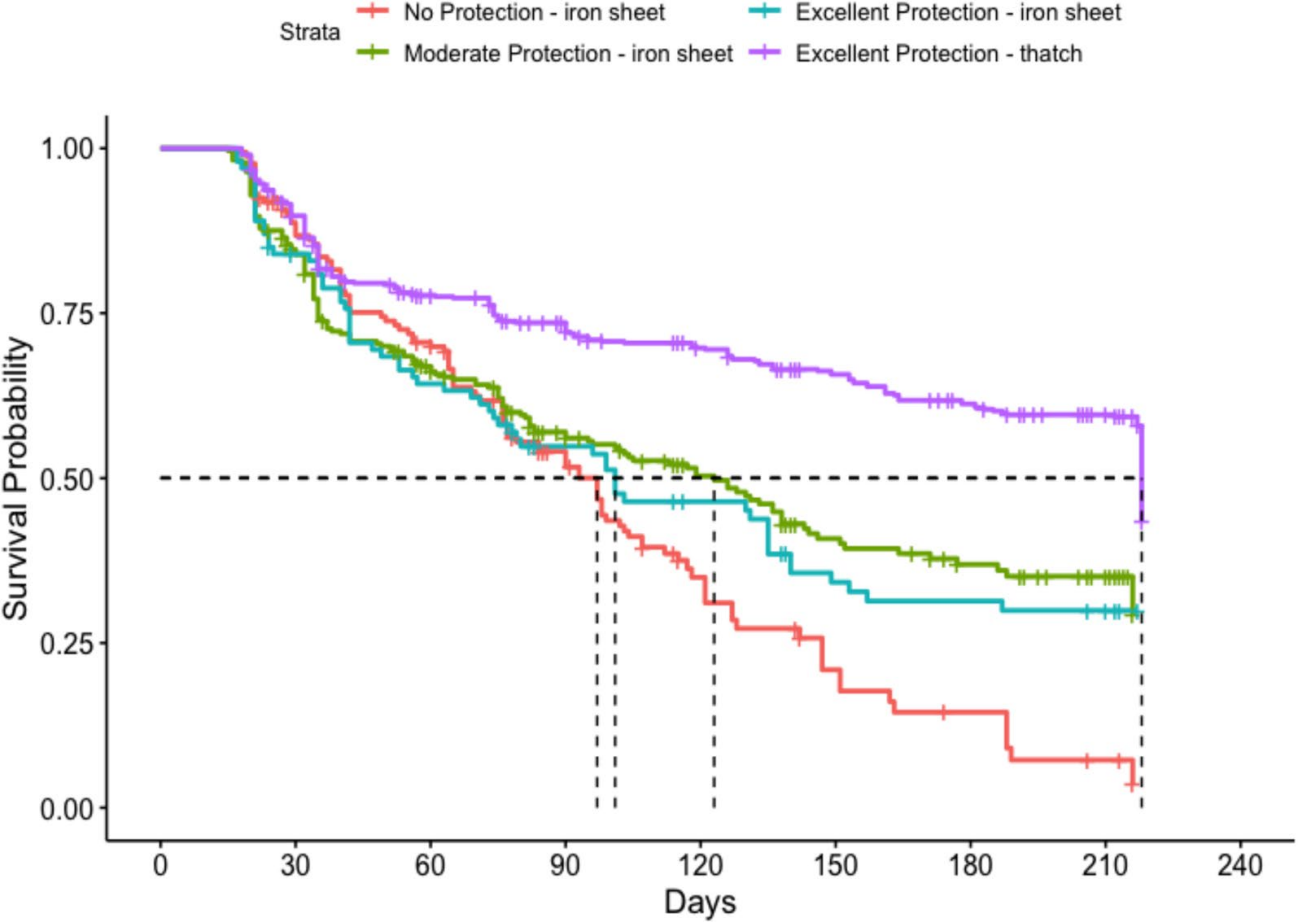
Kaplan–Meier curves of ATSB median survival times of roof labels by protection levels

**Fig. 5:**
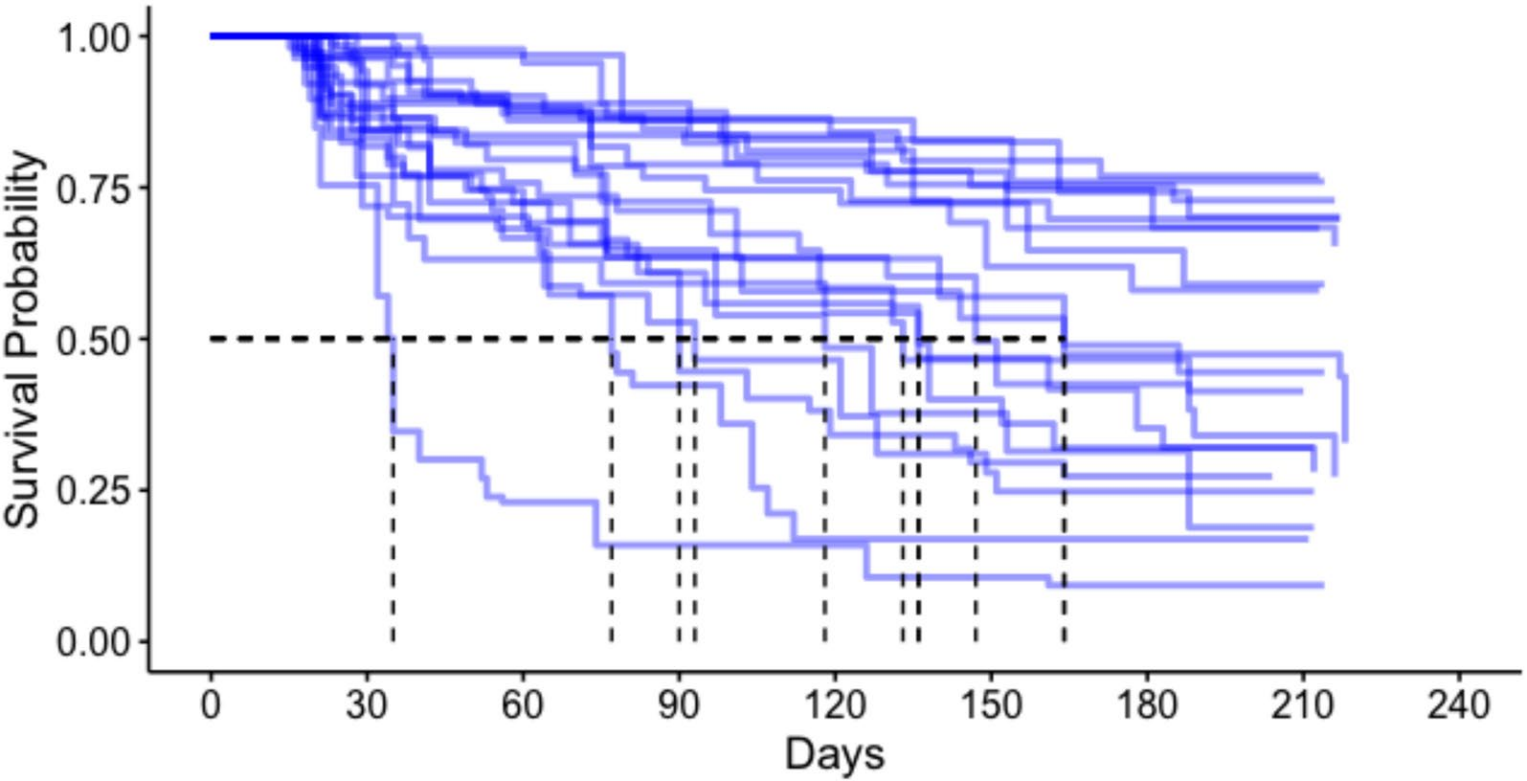
Kaplan–Meier curves of ATSB survival time in days in each of the 20 clusters

The median survival for all ATSB stations in this study was 149 days (95% CI 133, 164) (Fig. 2), approximately five months. ATSB stations hung on structures with thatch roofing had the longest median survival time at > 218 days. ATSB station median survival differed according to protection level of the installation site, with median survival of ATSB stations in locations with “excellent protection” of > 218 days, while median survival time in sites with “some protection” and “no protection” were 124 days and 90 days, respectively (Fig. 3). Figure 4 illustrates the median survival times of ATSBs placed under different roofing materials and corresponding protection levels. ATSB stations under thatch roof with “excellent protection” showed the survival median time of > 218 days, while the median survival time of ATSB stations under the iron sheet roof with “excellent protection” was 100 days. Cement roofs with “moderate” and “no protection” and thatch roofs with “moderate protection” were not included in these calculations because of the small sample sizes. Large differences in median survival time were also observed between different clusters (Fig. 5).

The Cox-Proportional hazards model indicated that ATSB stations installed in locations with “excellent protection” had a longer survival time than those in locations with “no protection” (hazard ratio (HR) = 0.36, 95% CI 0.25–0.49, Table 5). ATSB stations in locations with “moderate protection” also had longer survival times compared to those with “no protection” (HR 0.62, 95% CI 0.47–0.82). Thatch roof was also associated with improved ATSB station survival (HR 0.37, 95% CI 0.26–0.47) compared to ATSB stations on structures with iron sheet roofs.

**Table 5:** Determinants of ATSB survival, estimated by univariate Cox-proportional hazards models with shared frailty for cluster

Variable	Unadjusted hazard ratio	95% CI	P-value
Protection level			
Reference: no protection (n = 194)			
Moderate protection (n = 292)	0.62	0.47, 0.82	0.001
Excellent protection (n = 621)	0.36	0.25, 0.49	< 0.001
Frailty (cluster)			< 0.001
Roof material			
Reference: iron sheet (n = 554)			
Cement or wood plank (n = 26)	1.32	0.79, 2.22	0.29
Thatch (n = 527)	0.37	0.26, 0.47	< 0.001
Frailty (cluster)			< 0.001
Distance of ATSB to ground			
Reference < 100 cm (n = 167)			
> 200 cm (n = 11)	5.28	2.13, 13.12	< 0.001
151–200 cm (n = 296)	1.84	1.32, 2.56	< 0.001
100–150 cm (n = 633)	1.35	1.04, 1.76	0.025
Frailty (cluster)			< 0.001

Shorter distance between the ATSB station and the ground was associated with improved longevity of the ATSB stations. The univariate Cox-Proportional Hazard models (Table 5) indicated that ATSB stations hung over 200 cm above the ground were more likely to be lost or damaged compared to those ATSB stations placed 100 cm or less above the ground (HR 5.28, 95% CI 2.13–13.12). ATSB stations installed at heights of 100–150 cm or 151–200 cm above the ground also had reduced survival compared to those placed at 1 m or lower (HR 1.35 and 1.84, respectively).

Including calendar month as a time-varying covariate in Cox-Proportional Hazard models indicated that ATSB survival was associated with time (Table 6). ATSBs were more likely to be damaged during the months of January (HR 1.75, 95% CI 1.23–2.48) and February (HR 1.38, 95% CI 1.01–1.90) than in November, while ATSBs were less likely to be damaged toward the end of the seasonal deployment period in May (HR 0.53, 95% CI 0.36–0.83). When including protection level alongside month in a multivariate model, a similar pattern remained with ATSBs more likely to be damaged in January and less so in May. There was evidence for an interaction between protection level and calendar month; however, sensitivity analysis indicated that this was driven by very high damage rates reported in one cluster.

**Table 6:** Determinants of ATSB survival, determined by Cox-Proportional Hazards models with shared frailty for cluster and time-varying covariate

	Univariate model			Multivariate model		
	Hazard ratio	95% CI	p-value	Hazard ratio	95% CI	p-value
Month						
November	Ref.	–	–	Ref.	–	–
December	1.04	0.74, 1.46	0.832	0.96	0.69, 1.35	0.834
January	1.75	1.23, 2.48	0.002	1.57	1.11, 2.23	0.010
February	1.38	1.01, 1.90	0.045	0.22	0.89, 1.68	0.225
March	0.80	0.56, 1.15	0.237	0.71	0.49, 1.02	0.063
April	0.88	0.61, 1.26	0.479	0.75	0.52, 1.08	0.120
May	0.53	0.34, 0.83	0.005	0.45	0.29, 0.71	0.001
Protection level						
None	–	–	–	Ref.	–	–
Moderate	–	–	–	0.61	0.24, 0.46	0.001
Excellent	–	–	–	0.34	0.46, 0.80	< 0.001

## Discussion

This is the first study to estimate the survival time of an ATSB product during seasonal deployment to control malaria in Africa. ATSB stations installed with excellent protection had median survival times > 218 days, which is longer than the seasonal deployment period in this setting. The overall median survival time for ATSB stations was 149 days, meaning 50% of the ATSB stations were still hanging and in good condition after approximately five months. For comparison, the seasonal ATSB deployment period in this Zambian setting was approximately seven months (deployment in November and removal in June). ATSB stations may be appropriate for areas with seasonal malaria, such as in this Zambia setting, particularly if installed on structures offering a good level of protection, such as those with mud walls and thatch roofs.

In general, the protection level at which ATSB stations were installed was largely determined by the architectural style of the structure where they were hung. With roof type alone being a better predictor of survival time than protection level, though both protection level and architectural features showed strong correlations with survival time. The most common reasons for ATSB station replacement were holes/tears, mold growth, and leaking bait. These results are consistent with data from ATSB intervention monitoring implemented during the main trial in which this sub-study was conducted [10]. Anecdotally, leaks of ATSB contents tended to occur early in the transmission season, probably due to heavy rains in November–December. Findings published in Kyomuhangi et al. [10] support this; and indicate that most leaks occur at the start of the monitoring period. Calendar month was shown to influence ATSB survival, with increased hazard in January and reduced hazard in May compared to November. These findings support the hypothesis that heavy seasonal rains in January are associated with increased ATSB damage.

Large differences in the survival of ATSB stations were observed among the different clusters. While these differences may be partly attributable to differences in housing style between clusters, it is also likely that other environmental, structural, or socioeconomic differences contributed to variations in the longevity of the ATSB stations between clusters. In one cluster, a majority of the devices failed within the first 60 days, most damage in this area was believed to be caused by rodents, likely due to presence of farming areas near households. According to Kyomuhangi et al*.* [10], damage due to holes/tears led to a high turnover of ATSB stations on dwelling structures, where replacements for ATSB stations experiencing this damage type were themselves more likely to acquire holes/tears. It is plausible that rodent infestation was significant where housing structures are rudimentary with mud-brick walls and thatch roofing, hence contributing to the repeat damages and decline in survival probability of ATSB devices.

The use of a shared frailty model for the estimation of associations with survival is likely necessary to account for correlation in frailty [23] of individual ATSB stations within study clusters that share characteristics (including environmental exposures, foraging animals, weather elements, community cultural practices); however, additional clustering at the structure and household level was not accounted for in the analyses. Nevertheless, it is also important to recognize that survival or durability of vector control products such as ATSB, ITN, and IRS may vary greatly, even across small distances, due to differences between household and community characteristics.

As this study used a convenience sample of households and structures for enrolment, the characteristics of included households were not strictly representative of the wider ATSB trial area or of all of western Zambia. Given that housing characteristics are an important determinant of ATSB survival time, future deployments of similar products will have to carefully consider local household characteristics when determining deployment strategy, as well as replacement needs and timelines.

Another limitation of this study was right-censoring for a large number of ATSB stations. The ATSB stations were removed from all households at the end of the study period, resulting in right-censoring of a substantial number of ATSB stations, especially among those deployed under thatch roofs and/or excellent levels of protection. It is possible that under these conditions the ATSB stations could remain intact for a much longer period of time resulting in substantially more benefits and lower replacement needs, or costs as compared to when ATSB stations must be deployed with little protection.

While this study was focused on ATSB stations’ physical integrity as defined by criteria that were used during the first large-scale deployment of an ATSB, the extent to which these criteria for replacement are associated with loss of ATSB efficacy, bio-efficacy, attractancy, or other potential concerns, including community acceptability, is not known. As such, it is likely that the durability of ATSB stations in field settings could be substantially longer or shorter than our estimates of physical integrity and attrition here, especially if the efficacy of stations is poorly coupled to our replacement criteria. Understanding whether stations continue to prove attractive to mosquitoes and bio-effective at killing mosquitoes over time and in various conditions will be critical to fully understanding the real durability of future ATSB products. It is also important to note that ATSB station deployment in the study setting was not associated with a statistically significant reduction in malaria incidence or prevalence [24]. As such, this study may serve mainly as a guide to methods for durability studies of future ATSB products, should a class and a product establish efficacy rather than providing direct advice on how to use this specific product.

## Conclusion

Median survival time was greater than the seasonal deployment period (~ 7 months) for ATSB stations that were installed in protected locations, though the median survival time for all ATSB stations in the study was shorter at approximately five months. The majority of Sarabi v1.2 ATSB stations deployed in this setting will remain intact for a seven-month seasonal deployment period if they are installed in locations protected from rain and wind. Appropriate protection includes placement underneath the overhang of a thatch roof, which was a common housing characteristic in this study setting. These results suggest that future deployments of similar ATSB stations must carefully consider the architecture of structures and houses in planned deployment areas when determining deployment strategies. Further research is needed to understand factors that influence the efficacy, and bio-efficacy of ATSB stations after deployment regardless of ATSB station physical condition.

## Data Availability

De-identified data are available from the corresponding author on reasonable request. Following publication of forthcoming secondary analyses of trial data, the deidentified trial dataset will be posted on a public repository.
